# Novel Targeted Nano-Parthenolide Molecule against NF-kB in Acute Myeloid Leukemia

**DOI:** 10.3390/molecules24112103

**Published:** 2019-06-03

**Authors:** Noureldien H. E. Darwish, Thangirala Sudha, Kavitha Godugu, Dhruba J. Bharali, Osama Elbaz, Hasan A. Abd El-ghaffar, Emad Azmy, Nahla Anber, Shaker A. Mousa

**Affiliations:** 1 Hematology Unit, Clinical Pathology Department, Mansoura Faculty of Medicine, Mansoura University, Mansoura 35516, Egypt; osamaelbaz@yahoo.com (O.E.); haabdelghaffar@yahoo.com (H.A.A.E.-g.); 2 The Pharmaceutical Research Institute, Albany College of Pharmacy and Health Sciences, Rensselaer, NY 12144, USA; Sudha.thangirala@acphs.edu (T.S.); Kavitha.godugu@acphs.edu (K.G.); Dhruba.bharali@acphs.edu (D.J.B.); shaker.mousa@acphs.edu (S.A.M.); 3 Clinical Hematology Unit, Mansoura University Oncology Center, Mansoura University, Mansoura 35516, Egypt; dremadazmy7@gmail.com; 4 Fellow of Biochemistry Emergency Hospital, Mansoura University, Mansoura 35516, Egypt; nahla.anber@yahoo.com

**Keywords:** AML, nanoparticles with antiCD44 and encapsulating parthenolide, targeted therapy, nanoparticles, poly lactide co-glycolide

## Abstract

The targeted nano-encapsulation of anticancer drugs can improve drug delivery and the selective targeting of cancer cells. Nuclear factor kappa B (NF-kB) is a regulator for different biological responses, including cell proliferation and differentiation. In acute myeloid leukemia (AML), constitutive NF-κB has been detected in more than 50% of cases, enabling leukemic cells to resist apoptosis and stimulate uncontrolled proliferation. We evaluated NF-kB expression in bone marrow samples from 103 patients with AML using quantitative real time polymerase chain reaction (RT-PCR) and found that expression was increased in 80.5% (83 out 103) of these patients with AML in comparison to the control group. Furthermore, overexpressed transmembrane glycoprotein (CD44) on leukemic cells in comparison to normal cells is known to play an important role in leukemic cell engraftment and survival. We designed poly lactide co-glycolide (PLGA) nanoparticles conjugated with antiCD44 and encapsulating parthenolide (PTL), a nuclear factor kappa B (NF-kB) inhibitor, in order to improve the selectivity and targeting of leukemic cells and to spare normal cells. In vitro, in leukemic cell lines Kasumi-1, KG-1a, and THP-1, proliferation was decreased by 40% (** *p* < 0.01) with 5 µM PLGA-antiCD44-PTL nanoparticles in comparison to the same concentration of free PTL (~10%). The higher uptake of the nanoparticles by leukemic cells was confirmed with confocal microscopy. In conclusion, PLGA-antiCD44-PTL nanoparticles improved the bioavailability and selective targeting of leukemic cells, thus holding promise as a drug delivery system to improve the cure rate of AML.

## 1. Introduction

Acute myeloid leukemia (AML) is associated with a high relapse rate and poor overall survival, even with up-to-date chemotherapeutic drugs. The overall leukemia incidence rate increased by almost 2% per year (2004–2013), driven primarily by AML; AML incidence increased from 3.4 (per 100,000) in 2004 to 5.1 in 2013. In 2017, more than 21,000 patients were diagnosed as de novo AML cases in the United States and early death was observed in about 50% of older patients with AML [1].

In spite of achievements in the survival for patients with AML, especially younger patients, AML long-term survival is still an area of active research. Recently, various studies have reported clones known as leukemic stem cells (LSCs) as the main cause of relapse and chemotherapeutic resistance [2]. LSCs are described as a small chemoresistant clones that have endless self-renewal and produces blast cells in huge numbers [3]. As a step forward to improving the long-term survival for patients with AML, LSCs’ identification and targeting remains a hopeful project. Furthermore, the identification of surface or biological markers that are specific to leukemogenesis will be important in order to attack the leukemic cells only and avoid any damage to normal hematopoietic cells [2].

The nuclear factor kappa B (*NF-κB*) plays an important role as a regulator for different biological responses, including cell proliferation, differentiation, and immunological responses [4]. Recently, there has been strong evidence to suggest that the aberrant activation of *NF-κB* has a role in different types of cancer that may develop due to the interaction of *NF-κB* with other signaling pathways [5]. Therefore, *NF-κB* is considered as a poor prognostic factor in different types of cancer. Constitutively activated *NF-κB* was shown to transcriptionally activate Bcl-2 and Bcl-XL (anti-apoptotic/prosurvival factors), protecting tumor cells from apoptotic stimuli and various chemotherapeutic agents [6,7]. On the other hand, the sensitivity of malignant cells to chemotherapeutic agents was shown to be increased by the inhibition of *NF-κB*, which may result from pro-apoptotic signal activation and pro-survival response suppression [8,9]. All of these observations demonstrate the importance of *NF-κB* as a therapeutic target [10].

Inhibition of *NF-kB* with parthenolide (PTL), a naturally occurring sesquiterpene lactone, for eradication of leukemic cells was investigated recently. PTL is used as an anti-inflammatory agent in the treatment of fever and rheumatoid arthritis [11,12], and it can stimulate apoptotic pathways through the inhibition of *NF-κB*, activation of *p53*, and increase in reactive oxygen species [13]. However, it‘s lack of solubility in water, and thus bioavailability, limits its potential as a drug. Researchers are trying to develop synthetic analogs that will be better absorbed; PTL and a PTL analog, dimethylamino-parthenolide, have been shown to be effective against leukemic cells while sparing normal HSCs [14].

In addition to using PTL as an NF-kB inhibitor, we also chose to focus on the transmembrane glycoprotein CD44, which plays an important role as a signaling receptor involved in myelopoiesis. Different abnormal CD44 isoforms have been found in many types of malignant cells [15]. CD44 variant expression was noticed to be more common for AML cells than for normal cells, which reflects the importance of antiCD44 as a promising receptor-targeted delivery system for different anti-AML drugs [15].

We have developed a targeted molecule strategy in order to attack CD44 and NF-κB by using nanoparticles (NPs) of less than 200 nm in size, depending on our previous established protocol with antiCD24 and CD49f against bladder and breast cancers, respectively [16,17]. Such a strategy might improve not only the delivery of the drug but also the selective targeting of leukemic cells while sparing the normal components of bone marrow. We started by studying the expression of *NF-κB* in patients with AML and correlating it with survival. Next, we did AML cell culture experiments using three AML cell lines (Kasumi-1, KG-1a, and THP-1) and evaluated the effect of different concentrations of free PTL and nano-antiCD44 encapsulating PTL against the cells.

## 2. Results

### 2.1. Patients’ Assessment for NF-κB Expression

We found that expression of *NF-κB* was increased in 80.5% of patients with AML (83 out of 103) and increased 2.3- to 69.0-fold in comparison to the control group. Out of the 103 patients, statistically significant expression of *NF-κB* was observed in patients with AML, with FLT-ITD (26- to 29-fold, 8 patients), del 5 (~15-fold, 6 patients), and del 7 (~20-fold, 4 patients) mutations.

**Figure 1:**
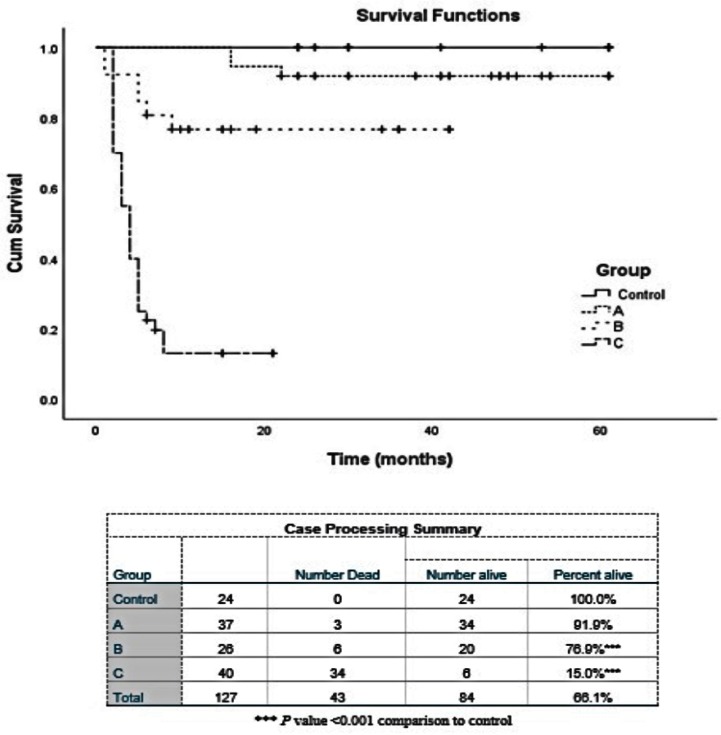
Kaplan-Meier survival analysis. Kaplan-Meier plots representing the correlation of *NF-κB* gene expression and survival. Group (A) < 5-fold increase, (B) 5-10 fold, (C) > 10 fold. Patients with low gene expression had longer survival time than those with high expression. For Kaplan-Meier plots, the log-rank test was applied. The log-rank test *p* value indicates the significance of the correlation.

The overall survival for the patients with AML indicates the presence of an inverse relationship between survival and *NF-κB* expression (e.g., longer survival was associated with patients with low gene expression and vice versa) (Figure 1). The worst prognosis was observed in patients with high *NF-κB* expression and FLT3-ITD mutation. On the other hand, a better prognosis was found in most of the patients with a mild increase in *NF-κB* expression (0.7- to 5.0-fold) and normal karyotyping. Further, the high expression of NF-κB in the 3 AML cell lines was confirmed with Western blot (data not shown).

### 2.2. Flowcytometry Study

**Figure 2:**
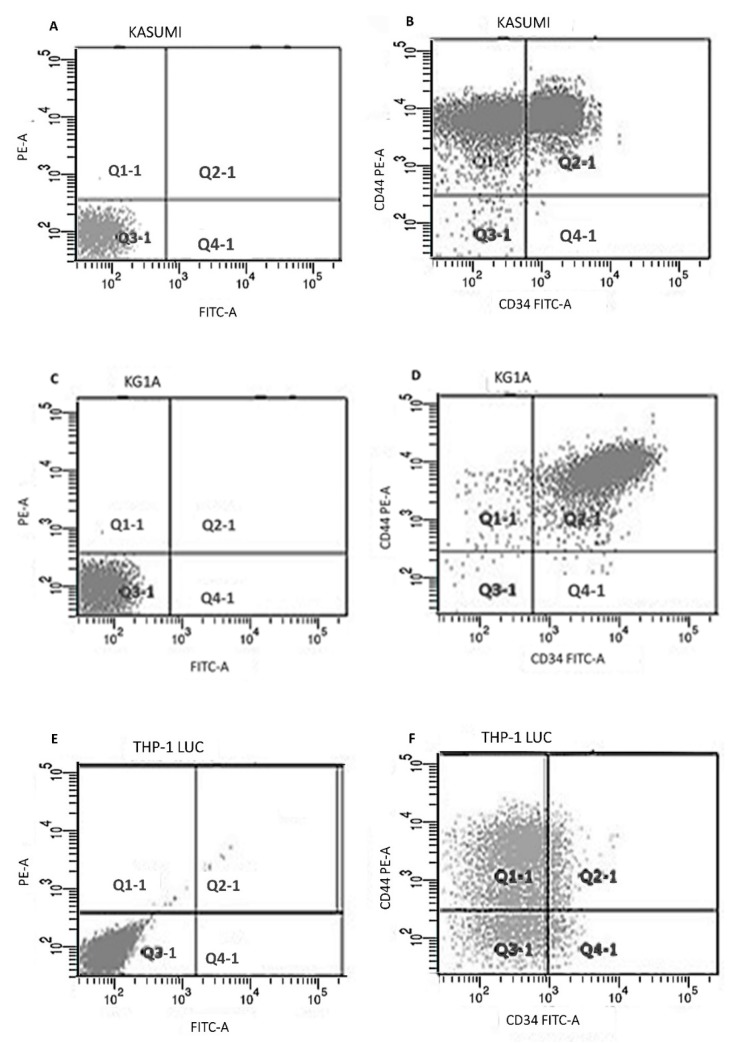
Flowcytometry analysis of CD34 and CD44expression in Kasumi-1, KG-1a, and THP-1 Lucia cell lines. (**A**,**C**,**E**) Isotype control for CD34 and CD44 expression in Kasumi-1, KG-1a, and THP-1 Lucia cell lines, respectively. (**B**,**D**,**F**) CD34 and CD44 expression in Kasumi-1, KG-1a and THP-1 Lucia cell lines, respectively.

The expression of CD34 and CD44 for the Kasumi-1, KG-1a, and THP-1 Lucia cell lines was analyzed using flowcytometry (Table 1). All Kasumi-1 cells express CD44, while CD34 expression was around 60% (Figure 2A,B). Almost all the KG-1a cells expressed CD34 and CD44 (Figure 2C,D). For THP-1, about 78% of the cells expressed CD44, while CD34 was 21% (Figure 2E,F).

### 2.3. Nanoparticle Characterization

**Figure 3:**
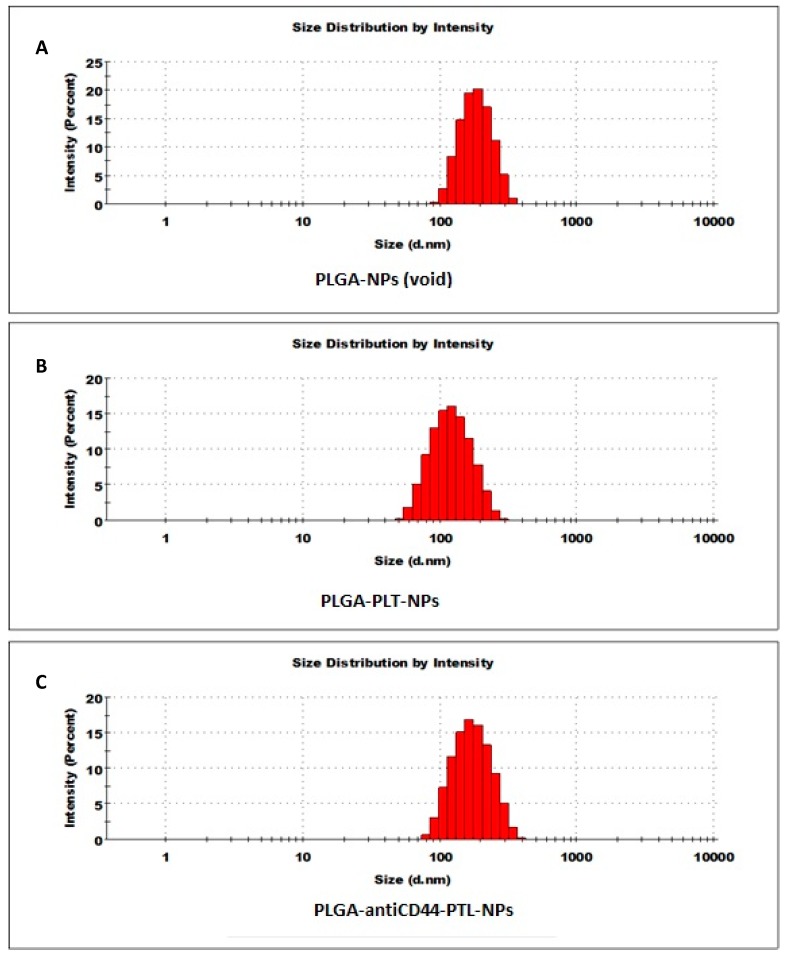
Size measurement of nanoparticles using Dynamic Light Scattering (DLS). (**A**) Void PLGA nanoparticles (PLGA-NPs). (**B**) PLGA nanoparticles encapsulating parthenolide (PLGA-PTL-NPs). (**C**) PLGA nanoparticles with antiCD44 and encapsulating parthenolide (PLGA-antiCD44-PTL-NPs). Abbreviations: NP, nanoparticle; PLGA, poly lactide co-glycolide; PTL, parthenolide.

**Figure 4:**
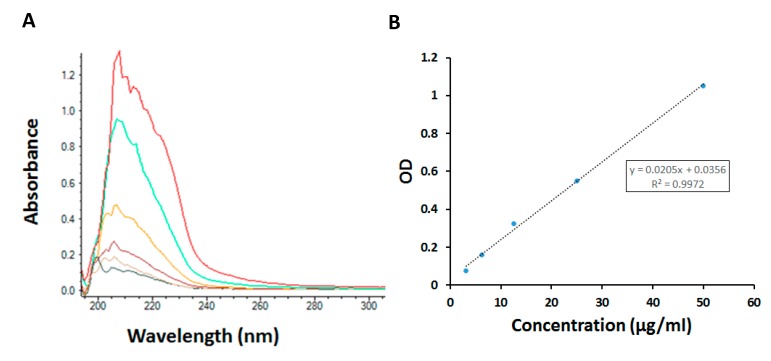
Determination of amount of parthenolide (PTL) encapsulated in poly lactide co-glycolide (PLGA) nanoparticles. (**A**) UV-VIS spectra used to construct the standard curve (**B**), with concentrations of PTL of 1.6, 3.2, 6.25, 12.5, 25.0, and 50.0 µg/mL.

The sizes, size distributions and zeta potentials of the different NPs synthesized for these studies are listed in Table 2. In addition, the DLS histogram showing the Z average size of the NPs is shown in Figure 3. The average size of the NPs was in the range 147 to 172 nm in diameter. The entrapment efficiency of PTL in the NPs was 65% and the loading efficiency was 4.2 mg PTL per 100 mg NPs, or 4.2% *w*/*w* (Figure 4).

### 2.4. Cell Vitality Assay

The three AML cell lines were used to evaluate the effect of targeting of NF-κB with free PTL and PLGA-antiCD44-PTL-NPs on the cell proliferation and to determine the optimal concentration. Identification of the ideal cell count and duplication time for each cell line using cell proliferation assay was important before running the MTT assay. A cell count of 100 × 10^3^/well and a duplication time of about 48 hrs were found to be ideal for the three cell lines. Different concentrations were used to evaluate the proper concentration that would interfere with cell proliferation.

**Figure 5:**
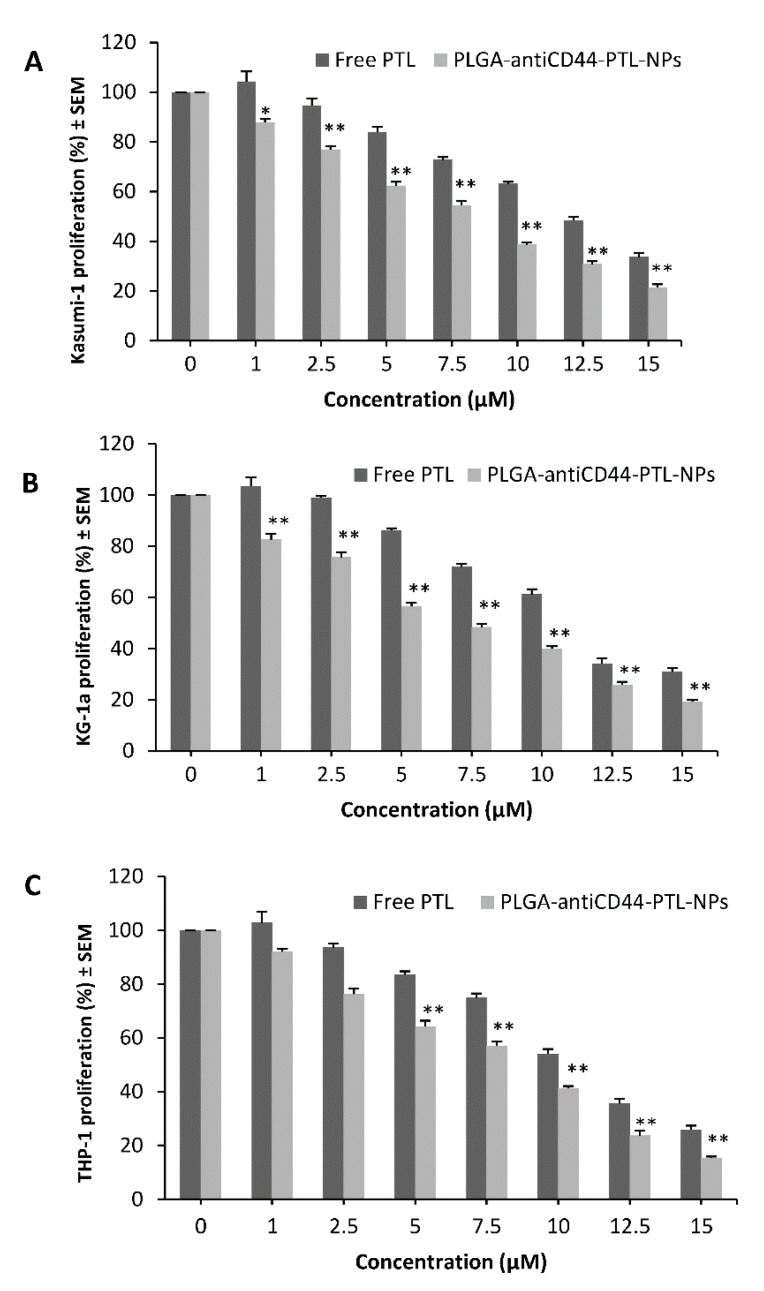
Cell lines’ proliferation with free parthenolide (PTL) and PLGA-antiCD44-PTL-NPs measured with MTT assay. (**A**) Kasumi-1 cell line, (**B**) KG-1a cell line, (**C**) THP-1 Lucia cell line. Shown with free PTL and PLGA-antiCD44-PTL-NPs at 1.0, 2.5, 5, 7.5, 10, 12.5, and 15 µM. Cell proliferation (% of control) is expressed as mean ± S.E.M., *n* = 3. One-way ANOVA was used followed by the Newman-Keuls post-test (* *p <* 0.05, ** *p <* 0.01 compared to free PTL). Abbreviations: NP, nanoparticle; PLGA, poly lactide co-glycolide; PTL, parthenolide.

In the 48 hrs MTT assay, Kasumi-1 (Figure 5A) and KG-1a (Figure 5B) showed 13% (*p* < 0.05) and 17% (*p* < 0.01) decrease in proliferation with 1 µM PLGA-antiCD44-PTL-NPs (the lowest concentration) in comparison to the same concentration of free PTL. For the THP-1 Lucia cell line, the 48 h MTT assay showed that 5 µM PLGA-antiCD44-PTL-NPs were associated with a 35% (*p* < 0.01) decrease in proliferation (Figure 5C).

We have tested all of the components separately and in combinations (antiCD44, PLGA, and antiCD44-PLGA without PTL), and we didn’t find any effect on the proliferation (data not shown).

### 2.5. Assessment of Cellular Uptake of PLGA-antiCD44-PTL-NPs 

**Figure 6:**
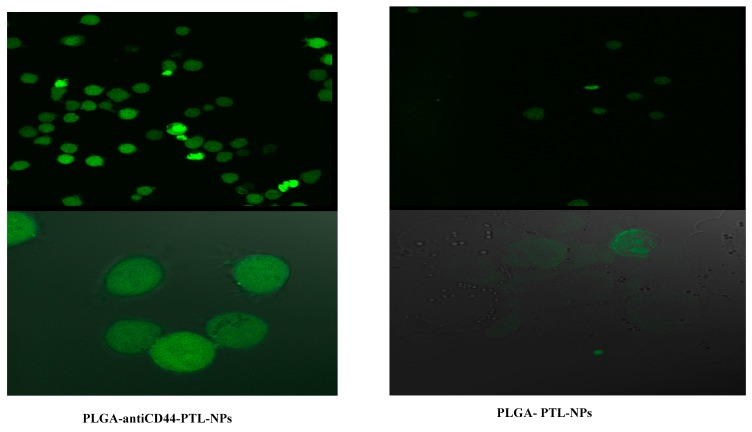
Confocal microscopy to assess nanoparticle uptake. KG-1a cells after incubation for 3 hrs with PLGA-antiCD44-PTL-NPs and PLGA-PTL-NPs (20X and 40X magnification power corresponding to upper and lower images, respectively). There was higher uptake of PLGA-antiCD44-PTL-NPs in comparison with PLGA-PTL-NPs by leukemic cells. Abbreviations: NP, nanoparticle; PLGA, poly lactide co-glycolide; PTL, parthenolide.

We compared the effectiveness of PLGA-antiCD44-PTL-NPs with PLGA-PTL-NPs using confocal microscopy. For both delivery systems, the nano shell (PLGA) was tagged with fluorescent FTIC. Photographic and fluorescent images showed greater uptake of PLGA-antiCD44-PTL-NPs in comparison to PLGA-PTL-NPs by leukemic cells (Figure 6).

## 3. Methods

### 3.1. Patient and Control Samples

Written informed consent was obtained from patients and controls after approval of the study protocol by the Local Ethical Committee, IRB (Institutional Research Board, Mansoura Faculty of Medicine, Mansoura University, R.18.03.76). Bone marrow samples of 103 Egyptian patients ranging in age from 17 to 79 years (average 42 ± 16.1 years), 47 (45.6%) males and 56 (54.3%) females, presenting with CD34+ and CD34- AML were obtained from newly diagnosed patients with AML. The exclusion criteria included receiving therapies for any malignancies and the presence of myelodysplastic features. Diagnoses of patients were established on bone marrow smear examination, cytochemistry, flow cytometry, and cytogenetics. The control group was represented by normal bone marrow samples obtained from 24 patients before undergoing splenectomy surgery, and they ranged in age from 24 to 61 years (average 43.75 ± 15.72 years), 12 (50%) males and 12 (50%) females. Gene expression studies compared samples from patients with AML to the control samples.

### 3.2. Isolation of Mononuclear Cells (MNCs) from Bone Marrow Samples

Patient and control samples obtained through bone marrow aspirate (5-10 mL) were collected with heparin (anticoagulant). The samples were rapidly prepared using a Ficoll gradient (1.077 g/mL) (Amersham Biosciences, Freiburg, Germany) and subsequent red blood cell lysis. Cells were then frozen in RPMI 1640 with 20% heat inactivated fetal bovine serum (Sigma, Saint Louis, MO, USA) and 5% DMSO (Riedel-de Haen, Seelze, Germany) in isopropanol-filled containers and subsequently stored in liquid nitrogen. When needed for analysis, cells were thawed and centrifuged to remove the supernatant and the pellet was used.

### 3.3. RNA Extraction and Quantitative RT-PCR

RNA extraction was performed using a TRI kit (Sigma), according to the kit‘s instructions, using the bone marrow mononuclear cells (MNCs) of both the patients and control groups. Before running qPCR, the same RNA concentration was established for all samples. 

For the gene expression study, quantitative PCR was performed in triplicate for both the patient and control groups using Realplex Sequence Detection System (Eppendorf, Hauppauge, NY, USA) Step PlusOne thermal cycler (Thermo Fisher Scientific, Waltham, MA) with the following setting: 95 °C for 10 min, followed by 40 cycles of 95 °C for 15 sec, and then 60 °C for 1 min. The comparative CT (2-ΔΔCt) method was used to study the relative gene expressions in both groups. We used *NF-κB*, and *HTRP* (housekeeping gene, used as internal control) primers from Invitrogen (Grand Island, NY, USA) (Table 3).

### 3.4. Cell Culture Experiments

#### 3.4.1. Cell Lines

For the in vitro study, three cell lines (Kasumi-1, KG-1a, and THP-1 Lucia) from the American Type Culture Collection (ATCC, Manassas, VA, USA) were used. These cell lines are commonly used in research studies as AML cell lines [18,19].

Kasumi-1 and KG-1a represented acute myelocytic leukemia. The Kasumi-1 cell line is associated with t(8;21). This translocation gives rise to the fusion gene *AML1-ETO* (also known as *Runx1-CBF2T1*). The resulting fusion protein AML1-ETO plays an important role in the downregulation of CEBPA mRNA, protein and DNA binding activity, leading to great disturbance in granulocytic differentiation [20]. The KG-1a cell line is associated with del (7), which was found in only about 50% of the metaphases [21]. THP-1 cells represent the t(9;11), which is associated with acute monocytic leukemia [22].

#### 3.4.2. Cell Vitality and Morphology

Blast cells from the three cell lines were assessed every two days using Leishman’s stain (Sigma, St. Louis, MO, USA) in order to assess maturation, number and morphology of myeloblasts. Trypan Blue (Life Technologies, Grand Island, NY, USA) was used to assess the vitality of myeloblasts every two days also. Kasumi-1, KG-1a, and THP-1 cell lines showed no differentiation or maturation over 10 passages. 

#### 3.4.3. Flow Cytometry Analysis

Fresh cells were incubated with monoclonal antibodies for 15 min at room temperature, washed once in PBS, and analyzed with flow cytometry. Monoclonal antibody combinations contained fluorescein isothiocyanate (FITC), phycoerythrin (PE), and allophycocyanin (APC). Anti-CD34 (FITC), Anti-CD44 (PE), Anti-CD45 (APC), and Hoechst 33342 were all from BD Biosciences (San Jose, CA, USA). For the 3 cell lines, the main combination was CD34/CD44/CD45/Hoechst 33342. Data acquisition was performed using a FACS Aria III (BD Biosciences) equipped with an argon and red diode laser, and analysis was performed using Cell Quest software (BD Biosciences). Blasts were identified by CD45dim/low side scatter characteristics according to Vial and Lacombe [23]. All analyses were performed in duplicate.

### 3.5. Protein Analysis (Western Blot)

Whole-cell protein extracts were obtained from frozen cell pellets available from the Kasumi-1, KG-1a, and THP-1 Lucia cell lines, loaded onto a 10% SDS-polyacrylamide gel, and electroblotted to a PVDF membrane (Bio-Rad, Hercules, CA, USA). Blocked membranes were incubated sequentially with the monoclonal NF-κB antibody (Santa Cruz Biotechnology, Dallas, TX, USA), anti-mouse, and detected with enhanced chemiluminescence (Thermo-Scientific, Grand Island, NY) according to the manufacturer’s recommendations. Western blot analysis identified specific bands of 100 kDa and 50 kDa molecular weight corresponding to NF-κB P105 and NF-κB P50, respectively. 

### 3.6. Synthesis and Characterization of Nanoparticles

Polyvinyl alcohol (Moliwol 488), ethyl acetate, Traut’s reagent, and cellulose dialysis tubing were purchased from Sigma. Poly (lactide co-glycolide) (PLGA) was purchased from Evonik Industries (Birmingham, AL, USA). PTL was purchased from Selleckchem (Houston, TX, USA). Purified monoclonal Ab CD44 (pan-CD44 antibody) was purchased from BD Bioscience.

PLGA NPs encapsulating PTL (PLGA-PTL-NPs) were synthesized by modifying a method originally developed in our laboratory [16,17,24,25,26,27]. Briefly, 20 mg of PTL and 200 mg of PLGA were dissolved in 2 mL ethyl acetate. To this solution, 12 mL of Moliwol 488 (2% *w*/*v* in DI water) was added and stirred with a magnetic stirrer for about 30 min. The solution was then sonicated intermittently for 90 sec with a QSonica probe sonicator (model CL-188, Newtown, CT, USA) (amplitude (μm) = 120; tip diameter of the probe 1/4” (6mm); voltage: 110 V, 50/60 Hz). Subsequently, the entire solution was stirred for another 2–3 h with the magnetic stirrer. Finally, the solution was dialyzed using dialysis membrane of 12–14 KDa cutoff, for about 12 h in order to remove ethyl acetate and free PTL. For the synthesis of void NPs (without PTL encapsulation, PLGA-NPs), the same steps were followed except no PTL was added in the first step.

Synthesis of PLGA antiCD44 NPs encapsulating PTL (PLGA-antiCD44-PTL-NPs) involved preparation of a solution containing 160 mg PLGA, 40 mg PLGA-maleimide, and 20 mg PTL in 2 mL of ethyl acetate. To this solution, 12 mL of Moliwol 488 (2% *w*/*v* in DI water) was added and stirred for about half an hr. This solution was then sonicated intermittently for about 90 sec and subsequently stirred with a magnetic stirrer for 2–3 h. Ethyl acetate and free PTL were removed with dialysis. Finally, antiCD44 was conjugated to the NPs encapsulating PTL with thiolated antiCD44 using Traut’s reagent [28]. The thiolated antiCD44 readily reacts with the maleimide group present on the NPs. We added 10 µg of the antiCD44 per 200 mg PLGA polymer for the nanoformulation, with the final concertation of antibody in the NP solution of around 0.06 µg/mL. For the synthesis of PLGA-antiCD44-NPs (without PTL encapsulation), the same steps were followed except no PTL was added. 

We used the same solvent for both the standard and unknown samples (NPs). Baseline correction was performed to cancel the absorption from the solvent. Thus, for each of the UV-spectra, baseline correction was done using the same solvent alone (without PTL). 

Dye labeled NPs were synthesized in a similar way, except in the first step additionally 10 mg of PLGA conjugated to FITC was added along with the PLGA and PLGA-maleimide.

In solution form, we have seen that these NPs are stable for at least two months. However, when lyophilized they can be stable for more than one year in powdered form.

#### 3.6.1. Dynamic Laser Light Scattering (DLS)

The sizes and size distributions of void NPs, PLGA-PTL-NPs, and PLGA-antiCD44-PTL-NPs in aqueous dispersions were determined using a Malvern zeta sizer (Malvern Instrumentation Co, Westborough, MA, USA). One ml of a NP solution was pipetted into a 3 mL, four-sided, clear plastic cuvette and measured directly. 

#### 3.6.2. Determination of PTL Amount in Nanoparticles

In order to determine the amount of PTL in the NPs, first the NPs were disintegrated and then the amount of PTL was determined using a UV-Vis spectrophotometer (Nanodrop 2000C spectrophotometer, Thermo Fisher Scientific). Absorbance was measured at 210 nm. The entrapment efficiency was determined using the following formula:

Entrapment efficiency = ([PTL]_f_)/([PTL]_t_) × 100 (1)

where [PTL]_f_ is the amount of PTL in the NPs and [PTL]_t_ is the theoretical amount of PTL (= total amount of PTL added initially). 

#### 3.6.3. Fluorescence Intensity

The fluorescence intensity of the dye labeled NPs was assessed with confocal microscopy, (Leica TCS SP5, Wetzlar, Germany). Photographic and fluorescent images were taken at constant exposure time for evaluation of intensity and NP uptake by leukemic cell lines. Cells were imaged at an excitation wavelength of 488 nm; emission was detected between 505 nm and 560 nm.

### 3.7. Cell Vitality Assay (MTT Assay)

The cell vitality assay was performed in triplicate using the 3 AML cell lines at a concentration of ~100 × 10^3^ per well (96-well plates). The cells were treated with PTL and PLGA-antiCD44-NPs (for encapsulating PTL) using seven different concentrations for each: 1, 2.5, 5, 7.5, 10, 12.5 and 15 µM. The suspended cells’ vitality was evaluated with the MTT assay over 48 h.

### 3.8. Statistical Analysis

Data were analyzed using GraphPad InStat 3 (GraphPad, San Diego, CA, USA). The analysis of variance (ANOVA) test was followed by Newman-Keuls to compare between experimental groups. Kaplan-Meier plot analysis and the log-rank test were used when survival was evaluated. The log-rank test *p* value indicates the significance of the correlation. Statistical significance is defined as * *p* < 0.05, ** *p* < 0.01. All results are expressed as means ± S.E.M.

## 4. Discussion

Bone marrow samples from patients with AML and the control group were preferred over cell lines for the *NF-κB* gene expression study. These samples are known to be richer in stem cells and early immature hematopoietic cells compared to peripheral blood. The selection of Kasumi-1, KG-1a, and THP-1 Lucia cell lines was related to the properties of these cells including the inability for spontaneous differentiation and unresponsiveness to colony stimulating factor.

### 4.1. NF-κB and Acute Myeloid Leukemia

Under the inactivated condition, inhibitor of kappa-B (I-κB) masks the nuclear localization signal of NF-κB, sequestering the NF-κB complexes in the cytoplasm [29]. On activation of NF-κB, I-κBs are phosphorylated by a protein known as the I-κB kinase complex at specific amino acid sequences (Ser-32 and Ser-36 of I-κBα; Ser-19 and Ser-23 of I-κBβ), and NF-κB is no longer inhibited by I-κB [30]. The released NF-κB can translocate to the nucleus, leading to activation of several transactivate κB-responsive elements [31].

The relation between *NF-κB* activation and cell proliferation over cell-cycle arrest appeared to be based on the relative balance between NF-κB’s biological and biochemical functions [32]. In addition to the important role of *NF-κB* in resistance of apoptosis and controlling the division of hematopoietic stem cell, it has recently become well-defined that *NF-κB* also has roles in oxidative stress [8]. *NF-κB* activation is responsible for inducible nitric oxide synthase (iNOS) activation to increase nitric oxide (NO), which has been described as a pro-apoptotic function of *NF-κB* [33,34,35]. The pattern of NO production may control cell survival because it was found that the acute production of NO triggers apoptosis, but on the other hand, the chronic production of NO by constitutively active *NF-κB* signaling could inhibit the apoptosis mechanism [36].

The upregulation of various *NF-κB* target genes has been reported in different types of cancer tumors. Among these, inhibitors of apoptosis (IAPs), FLICE-like inhibitory protein (FLIP), and some members of the anti-apoptotic Bcl-2 family inhibit apoptosis [37,38,39]. In addition, the activation of *NF-κB* associates with the upregulation of cell proliferation enhancers (e.g., cyclin D1 and c-myc), cell adhesion molecules, and several angiogenesis factors enhance cancer cell engraftment (e.g., ICAM-1 and VEGF) [37,38,39,40,41,42,43].

Finally, *NF-κB* activation is responsible for the regulation of heme oxygenase-1 (HO-1) expression, which is a well-known catabolizing enzyme for the free heme [44]. HO-1 has a protecting role against apoptosis by enhancing free heme catabolism, which causes damage in lipid bilayers of cell membranes [45]. The upregulation of HO-1 has been reported in AML and contributes to evading tumor necrosis factor-α (TNF)-induced apoptosis [46] as well as to chemotherapy-induced apoptosis [47]. The downregulation of *NF-κB* will impair the long-term expansion and self-renewal of leukemic cells and that will be an attractive approach for anti-AML therapy.

Here, we demonstrated that *NF-κB* was significantly increased in patients with AML, in particular those with molecular abnormality (e.g., FLT-3 ITD), while a moderate increase was observed in patients with AML with normal cytogenetics in comparison to the control group. This increase in *NF-κB* expression may be related to the NF-κB function as a transcription factor, which can explain the increase of leukemic cells’ division.

Consistent with our results, Guzman et al. reported the high expression of *NF-κB* in CD34+ AML cells but not in CD34+ normal hematopoietic cells in electrophoretic mobility shift assays [48]. Also, Baumgartner et al. reported a higher level of *NF-κB* activity in both de novo and relapsed patients with AML (35 patients) compared with controls with no correlation between CD34+ and CD34- blasts [49].

Cilloni et al. reported that more than 50% of analyzed patients with AML showed increased *NF-κB* activity. Those patients were associated with more aggressive features including higher white blood cell counts and blast cells in peripheral and bone marrow. That report suggested a possible link between the high expression of *NF-κB* and poor prognosis in patients with AML [50].

Interestingly, like our results in AML, Kordes and colleagues observed *NF-κB* activity in 39 of 42 acute lymphoblastic leukemia (ALL) specimens [51]. These results give the central role of NF-κB as a transcriptional regulator; expression of this factor in both AML and ALL cells represents a striking biologic distinction between leukemic and normal tissue.

Our study of the activity of *NF-κB* in primitive AML cells and the correlation with cytogenetic abnormality and patients’ survival appears to represent unique and previously undocumented data for this factor in the biology of leukemic cells.

### 4.2. NF-κB Targeting

PTL has a variety of reported in vitro biological activities, including suppression of NF-κB activity and increase of reactive oxygen species. The high expression of NF-κB within the blast cells and the leukemic stem cells might be used for the eradication of selective leukemic cells that could be regulated by the modulation of the *NF-κB* pathway [52].

NF-κB could be inhibited in leukemic cells by PTL, leading to decreased engraftment of leukemic cells, but the presence of NF-κB within the normal cell in variable amounts may cause some harm to these normal cells [53]. On the other hand, the use of nano encapsulation of PTL will improve the chance to target the leukemic cells and not harm normal cells.

Here, we tested cell proliferation in the presence of free PTL andPLGA-antiCD44-PTL-NPs in three leukemic cell lines. Our results reflect the improvement in targeting of the leukemic cells. This improvement related mainly to the antiCD44 incorporated in our NPs. Confocal microscopy confirmed the high uptake of PLGA-antiCD44-PTL-NPs in comparison with PLGA-PTL-NPs.

CD44 has a vital role not only in cell adhesion, but also in survival of progenitor cells, proliferation, differentiation, and migration. Malignant cells, and leukemic cells in particular, are represented with overexpression of CD44 in comparison to normal HSCs [54]. Here, we reported the high expression of CD44 in three AML cell lines, suggesting the important role of CD44 in both engraftment and cell signaling.

Consistent with our results, Guzman et al. demonstrated the ability of PTL to induce apoptosis in primary human AML cells and blast crisis chronic myelogenous leukemia cells while sparing normal hematopoietic cells. Eighteen-hour treatment with PTL at 7.5 μM was highly toxic to leukemic populations [55].

Steele et al. studied the in vitro actions of PTL on cells isolated from patients with chronic lymphocytic leukemia (CLL). A 3 h exposure to PTL was sufficient to induce apoptosis in CLL cells, and CLL cells were more sensitive to PTL than were normal T lymphocytes or CD34(+) hematopoietic progenitor cells [56].

Diamanti et al. showed that in vitro PTL-treated leukemic cells were associated with prevention or significantly reduced capacity for engraftment in the ALL mice model. They hypothesized that PTL can induce apoptosis in primitive and more differentiated leukemic cells and prevent disease establishment in vivo [13].

Sudha et al. found that encapsulating antitumor drugs like cisplatin, paclitaxel, or doxorubicin in PLGA was associated with increases in the tumor uptake of drug. The main function of PLGA encapsulation is the prolongation of the drug half-life in the circulation [24]. The main problem for PTL is its low water solubility, which may affect its bioavailability in vivo. Relevant to this, Sudha et al. also reported an increased uptake (~5-fold) of encapsulated cisplatin by the tumor compared with conventionally administered cisplatin [25]. Here, we found that the encapsulation of PTL with PLGA and antiCD44 may improve its bioavailability and uptake by leukemic cells only. Further studies are required to confirm the expression of these markers in a larger number of patients in correlation with the full chromosomal and molecular studies, especially to FLT3-ITD. Further, we are working on evaluating the effects of our target strategy in an AML mouse model as part of the target strategy of attacking both extracellular markers (CD44) and intracellular molecule (NF-κB) at the same time. Such a strategy might improve the selective targeting of AML blast cells and result in reduced bone marrow cytotoxicity.

## 5. Conclusions

In summary, we provided indicators of the correlation between the expression level of NF-κB and the prognosis in our patients with AML. Thus, NF-κB might be used not only as a prognostic marker but also as a possible marker for the selective targeting of leukemic cells and for eventually improving the cure rate of AML. One of the main obstacles in the treatment of a cancer such as AML is that chemotherapeutic agents destroy both cancer cells and at the same time some normal cells. In the last few years, nanotechnology has provided many effective strategies for the detection and treatment of cancer, overcoming the obstacles associated with conventional cancer diagnosis and therapy. Nanoparticle systems can be involved in many fields including the targeted in vivo delivery of imaging or treatment modalities to a tumor specifically.

## Figures and Tables

**Table 1:** Some stem cell markers expressed by Kasumi-1, KG-1a, and THP-1 Lucia cell lines expressed as percentages from the flow cytometry stem cell marker assay.

Cell Line	CD34 (%)	CD44 (%)	CD34/CD44 (%)
Kasumi-1	63	99	61
KG-1a	98	98	97
THP-1 Lucia	21	78	13

**Table 2:** Size, size distribution (polydispersity index PDI), and zeta potentials of the nanoparticles.

Nanoparticle	Size (nm)	PDI	Zeta Potential (ζ)
PLGA-NPs (void)	176	0.056	−13.6
PLGA-PTL-NPs	147	0.096	−15.2
PLGA-antiCD44-PTL-NPs	162	0.098	−15.8

Abbreviations: NP, nanoparticle; PLGA, poly lactide co-glycolide; PTL, parthenolide.

**Table 3:** Quantitative RT-PCR primers used in the TaqMan gene expression assay.

Gene Symbol	Gene Description	Assay ID	Amplicon Length
*RELA*	Nuclear factor kappa B (*NF-κB*)	Hs00428211_m1	87
*HPRT1*	Human hypoxanthine phosphoribosyl transferase 1 (house-keeping gene)	Hs02800695_m1	82
